# Bayesian modeling of ChIP-chip data using latent variables

**DOI:** 10.1186/1471-2105-10-352

**Published:** 2009-10-26

**Authors:** Mingqi Wu, Faming Liang, Yanan Tian

**Affiliations:** 1 Department of Statistics, Texas A&M University, College Station, TX 77843, USA; 2 Department of Veterinary Physiology and Pharmacology, Texas A&M University, College Station, TX 77843, USA

## Abstract

**Background:**

The ChIP-chip technology has been used in a wide range of biomedical studies, such as identification of human transcription factor binding sites, investigation of DNA methylation, and investigation of histone modifications in animals and plants. Various methods have been proposed in the literature for analyzing the ChIP-chip data, such as the sliding window methods, the hidden Markov model-based methods, and Bayesian methods. Although, due to the integrated consideration of uncertainty of the models and model parameters, Bayesian methods can potentially work better than the other two classes of methods, the existing Bayesian methods do not perform satisfactorily. They usually require multiple replicates or some extra experimental information to parametrize the model, and long CPU time due to involving of MCMC simulations.

**Results:**

In this paper, we propose a Bayesian latent model for the ChIP-chip data. The new model mainly differs from the existing Bayesian models, such as the joint deconvolution model, the hierarchical gamma mixture model, and the Bayesian hierarchical model, in two respects. Firstly, it works on the difference between the averaged treatment and control samples. This enables the use of a simple model for the data, which avoids the probe-specific effect and the sample (control/treatment) effect. As a consequence, this enables an efficient MCMC simulation of the posterior distribution of the model, and also makes the model more robust to the outliers. Secondly, it models the neighboring dependence of probes by introducing a latent indicator vector. A truncated Poisson prior distribution is assumed for the latent indicator variable, with the rationale being justified at length.

**Conclusion:**

The Bayesian latent method is successfully applied to real and ten simulated datasets, with comparisons with some of the existing Bayesian methods, hidden Markov model methods, and sliding window methods. The numerical results indicate that the Bayesian latent method can outperform other methods, especially when the data contain outliers.

## Background

The chromatin immunoprecipitation (ChIP) coupled with microarray (chip) analysis, provides the researchers an efficient way of mapping protein-DNA interactions across a whole genome. The ChIP-chip technology has been used in a wide range of biomedical studies, such as identification of human transcription factor binding sites, investigation of DNA methylation, and investigation of histone modifications in animals and plants [1-4]. Data from ChIP-chip experiments encompass DNA-protein interaction measurements on millions of short oligonucleotides (also known as probes) which often tile one or several chromosomes or even the whole genome. The data analysis consists of two steps: (1) identifying the bound regions where DNA and the protein are cross-linked in the experiments; and (2) identifying the binding sites through sequence analysis of the bound regions. The goal of this paper is to develop an effective method for the first step analysis.

Analysis of the ChIP-chip data is very challenging, due to the large amount of probes and the small number of replicates. The existing methods in the literature can be roughly grouped into three categories, the sliding window methods [1,5-7], the hidden Markov Model (HMM) methods [6,8-10], and the Bayesian methods [11-13]. Other methods have been suggested, e.g., by Zheng [14], Huber [15] and Reiss [16], but are less common.

The sliding window methods are to test a hypothesis for each probe using the information from the probes within a certain genomic distance sliding window, and then try to correct for the multiple hypothesis tests. The test statistics used are varied. Cawley [1] used Wilcoxon's rank sum test, Keles [7] used a scan statistic which is the average of *t*-statistics within the sliding window, and Ji and Wong [6] used a scan statistic which is the average of empirical Bayesian *t*-statistics within the sliding window. Since each test uses information from neighboring probes, the tests are not independent, rendering a difficult adjustment in the multiple hypothesis testing step. We note that the power of the sliding window tests is usually low, especially for the tests for the regions where the probe density is low. This is because there will be only very limited neighboring information available for those tests. Since, in the ChIP-chip experiments, the DNA samples hybridized to the microarrays are prepared by PCR, which is known to perform independently of the form of DNA, the far probes should have similar intensity patterns as long as they are of similar positions to their nearest bound regions. This provides a basis for us to devise powerful methods that make use of information from all probes.

The HMM methods have the potential to make use of all probe information, where the model parameters are estimated using all available data. However, in most of the existing implementations of HMMs, the model parameters are estimated in an *ad hoc *way. For example, Li [8] estimated the model parameters using previous results on Affymetrix SNPs arrays. An exception is tileHMM [10], where the model parameters are estimated using the Baum-Welch and Viterbi training algorithms [17]. However, as pointed out by Humburg et al. [10], the Baum-Welch algorithm tends to converge to a local maximum of the likelihood function, and the Viterbi training algorithm even fails to converge to a local maximum of the likelihood in some cases. This renders that the parameter estimates and thus followed inference often suboptimal to the problem.

Bayesian methods have also the potential to make use of all probe information. Like the HMM methods, the Bayesian methods estimate the model parameters using all available data. However, these methods usually require multiple replicates or some extra experimental information to parameterize the model. For example, the joint binding deconvolution model [11] requires one to know the DNA fragment lengths, measured separately for each sample via extrophoretic analysis; and the hierarchical gamma mixture model(HGMM) [12] requires one to first divide the data into genomic regions containing at most one bound region, but such information is, in general, unavailable. The Bayesian hierarchical model [13] models the probe intensities using essentially a mixture of normal distributions, and models the spatial structure of the probes using a Gaussian intrinsic auto-regression model [18]. Gottardo [13] developed a software for the model, Bayesian analysis of ChIP-chip (or BAC for short). Using BAC [13] does not need extra experimental information, but it is extremely slow, roughly 10 hours for a dataset with 300,000 probes on a personal computer. One reason for the slow speed is the use of MCMC simulations.

In this paper, we propose a Bayesian latent variable model for tiling array data. Our method differs from the existing Bayesian methods, such as the joint binding deconvolution model [11], the HGMM [12], and the Bayesian hierarchical model [13], in several respects. Firstly, it works on the difference between the averaged treatment and control samples. This enables the use of a simple model for the data, which avoids the probe-specific effect and the sample (control/treatment) effect. As a consequence, this enables an efficient MCMC simulation of the posterior distribution of the model, and also makes the model rather robust to the outliers. Secondly, it models the neighboring dependence of probes by introducing a latent indicator vector. Thirdly, it does not require multiple replicates or extra experimental information. As described below, it can work on a single intensity measurement for the probes. The Bayesian latent model has been successfully applied to several real and ten simulated datasets, with comparisons with some of the existing Bayesian methods, hidden Markov model methods, and sliding window methods. The numerical results indicate that the Bayesian latent model can outperform the others, especially when the data contain outliers. Our method is also computationally efficient; it takes about 30 minutes for a dataset with 300,000 probes on a personal computer.

The remainder of this paper is organized as follows. In Section 2, we describe the Bayesian latent model and its MCMC implementation. In Section 3, we test the Bayesian latent model on real and simulated datasets with comparisons with tileHMM, BAC and some sliding window methods. In Section 4, we discuss possible extensions of our methods and provide an explanation why our method outperforms tileHMM and BAC via a detailed comparison of the models used by them. In Section 5, we conclude the paper.

## Methods

Consider a ChIP-chip experiment with two conditions, treatment and control. Let *X*_1 _and *X*_2 _denote, respectively, the samples measured under the treatment and control conditions. Each sample has *m*_*l*_, *l *= 1, 2, replicates providing measurements for *n *genomic locations along a chromosome or the genome. Suppose that these samples have been normalized and log-transformed. In this paper, we summarize the measurements for each probe by(1)

where  is the intensity measurement of probe *i *averaged over *m*_*l *_replicates.

The underlying assumption for the summary statistic in (1) is that the intensity measurements for each probes has a variance independent of its genomic position. The rationale is that the DNA samples used in the experiments are prepared by PCR, which is known to perform independently of the form of DNA, and that the amount of the DNA samples provides the main sources for the variation of probe intensities. We note that a similar assumption has also been made in other Bayesian software, e.g., tileHMM [10]. Otherwise, *Y*_*i *_can be adjusted by its standard error to a shrinkage *t*-statistic [19] or an empirical Bayes *t*-statistic [6], depending on the estimate of the standard error. Note that both the adjustments are toward the constant variance of probes. Even with the adjustments, the Bayesian latent model developed in this paper can still work reasonably well, as the normality assumption approximately holds for the modified *t*-statistics.

### The Bayesian latent model

Suppose that the data consists of a total of *K *bound regions, and that region *k *consists of *n*_*k *_(*k *= 1,...,*K*) consecutive probes. For convenience, we call all the non-bound regions by region 0 and denote by *n*_0_, the total number of probes contained in all the non-bound regions, although the probes in which may be non-consecutive. Thus, we have . Let ***z ***= (*z*_1_,...,*z*_*n*_) be a latent binary vector associated with the probes, where *z*_*i *_= 1 indicates that probe *i *belongs to a bound region and 0 otherwise. Given ***z***, we can re-index (*y*_1_,...,*y*_*n*_), a realization of (*Y*_1_,...,*Y*_*n*_), by *y*_*kj*_, *k *= 0,...,*K*, *j *= 1,...,*n*_*k*_. Then *y*_*kj *_can be modeled as follows,(2)

where *μ*_0 _is the overall mean, which models the difference of sample effects (between the treatment samples and the control samples); *ν*_0 _= 0 and *ν*_*k *_> 0, *k *= 1,...,*K *accounts for the difference of probe intensities in different bound regions; ϵ_*kj*_s are random errors independently and identically distributed as *N*(0, *σ*^2^). In words, model (2) assumes that, conditioning on the latent vector ***z***, *y*_*kj*_s are mutually independent and also identically distributed within the same bound region. We are aware that for the tiling array data, the probe intensities tend to form a peak around the true binding site. Since, given ***z***, the order of probes is meaningless to us, the model (2) is appropriate if ignoring the order of the probes. We note that a similar assumption has also been used in the HGMM and HMM methods. Conditioning on ***z***, the likelihood of the model can be written as(3)

To conduct a Bayesian analysis for the model, we specify the following prior distributions for the model parameters:(4)

where *IG*(·,·) denotes an inverse Gamma distribution, *U*(·,·) denotes a uniform distribution, and *α*, *β*, *ν*_*min*_, *ν*_*max *_are hyperparameters. In this paper, we set *α *= *β *= 0.05, which form a vague prior for *σ*^2^; and set *ν*_*min *_= 2*s*_*y *_and *ν*_*max *_= max_*i *_*y*_*i*_, where *s*_*y *_is the sample standard error of *y*_*i*_. Different values of *ν*_*min*_, e.g., *s*_*y *_and 1.5*s*_*y*_, have also been tried in our simulations, and the results are similar. The sensitivity issue of the Bayesian latent model to the hyperparameters will be further discussed in Section 3. In addition, we assume that the latent vector ***z ***follows a truncated Poisson distribution,(5)

where *K*, denoting the total number of bound regions specified by ***z***, and is thus a function of ***z***; *λ *is a hyperparameter; *K*_max _is the largest number of bounded regions allowed by the model; and

which makes the prior (5) a proper distribution. The rationale behind this prior can be explained as follows. Since the length of each bound region is very short comparing to the chromosome or the whole genome, it is reasonable to view each bound region as a single point, and thus, following the standard theory of Poisson process, the total number of bound regions can be modeled as a Poisson random variable. Conditioning on the total number of bound regions, as implied by (5), we put an equal prior probability on all possible configurations of ***z***, i.e., assuming a non-informative prior for ***z***. The prior (5) penalizes a large value of *K*, where the parameter *λ *represents the strength of penalty. We do not recommend to use a large value of *λ*, as the number of true bound regions is usually small and a large value of *λ *will lead to discovery of too many false bound regions. Our experience shows that a value of *λ *around 0.01 usually works well for the ChIP-chip data. In this paper, we set *λ *= 0.01 in all simulations. The parameter *K*_max _is usually set to a large number. We set *K*_max _= 5000 in all simulations of this paper. As long as the value of *K*_*max *_has been reasonably large, increasing it further would have a negligible effect on simulations. Finally, we would like to point out that the bound region identification problem can also be viewed as a change-point identification problem that has been widely studied in statistics. For the change-point identification problem, the same truncated Poisson prior has been used for modeling the total number of change-points by many authors, see, e.g., Phillips and Smith [20], Dension et al. [21], Liang and Wong [22], and Liang [23].

If *ν*_1_,...,*ν*_*K *_∈ (*ν*_*min*_, *ν*_*max*_), combining the likelihood and prior distributions, integrating out *σ*^2^, and taking the logarithm, we get the following log-posterior density function(6)

otherwise, the posterior is equal to 0.

Due to the design of ChIP-chip experiments, it is obvious that the intensity measurements of the neighboring probes are positively dependent. To model this dependence, we use a latent indicator vector ***z***. This makes our model different from the existing models, such as the joint binding deconvolution model [11], the HGMM [12], and the Bayesian hierarchical model [13]. Both the joint binding deconvolution model and the Bayesian hierarchical model model the mean of probe intensities through the Gaussian random field (GMF), although their formulations may not be in the standard form of the GMF. Like the Bayesian latent model, the HGMM models the mean of probe intensities by a piece-wise constant function. The difference is that the HGMM requires one to first divide the data into genomic regions containing at most one bound regions, and thus it allows different non-bound regions to have different means. Considering the physical property of PCR, which performs independently of the form of DNA, allowing different non-bound regions to have different mean values may not be necessary.

### MCMC simulations

To simulate from the posterior distribution (6), we used the Metropolis-within-Gibbs sampler [24]; see Appendix for the details. Note that when a component of ***z ***is updated, the sum of square terms in the posterior density can be calculated in a recursive manner, and this simplifies the computation of the posterior density greatly.

### Inference of bound regions

Let *p*_*i *_= *P*(*z*_*i *_= 1|***y***) be the marginal posterior probability that probe *i *belongs to a bound region. Since the bound regions are expected to consist of several consecutive probes with positive IP-enrichment effects, the regions which consists of several consecutive probes with high marginal posterior probabilities are likely to be bound regions. To identify such regions, we follow Gottardo [13] to consider the joint posterior probability(7)

where *i *is the index of the probes, *w *is a pre-specified half-window size, and *m *is the minimum number of probes belonging to the bound region. As explained in Gottardo [13], the purpose of introducing the joint posterior probability is to remove the false bound regions, which usually consists of only few isolated probes with large enrichment effects. We found that the choice *w *= 5 and *m *= 5 works well in practice. This choice of *w *is consistent with the moving window size used in other work, such as Ji and Wong [6], Keles [12], and Gottardo [13]. The choice of *m *is chosen for robustness to false bound regions. It also reflects our belief that a bound region should consist of at least five consecutive probes with large enrichment effects.

Note that estimation of *ρ*_*i *_is trivial based on the samples simulated from the posterior distribution. The value of *ρ*_*i *_depends on a lot of parameters, such as *w*, *m *and the hyperparameters of the model. However, we found that the orders of *ρ*_*i *_are rather robust to these parameters. This suggests us to treat *ρ*_*i *_as a conventional testing *p*-value, and to control the false discovery rate (FDR) of the bound regions using a FDR control method, e.g., the empirical Bayes method [25] (Efron, 2004) or the stochastic approximation-based empirical Bayes method [26]. Both the methods allow for the dependence between testing statistics and an empirical determination of the density of the testing statistics.

Although a strict control of FDR is important to the detection of bound regions, it is not the focus of this paper. In this paper, we will follow other Bayesian methods, such as BAC, to simply set a cut- off value of *ρ*_*i*_. We classify probe *i *as a probe in bound regions if *ρ*_*i *_≥ 0.5, and classify probe *i *as a probe in nonbound region otherwise. As we will see in the numerical examples, the joint posterior probability can lead to a good detection of true bound regions.

## Results

### The Estrogen Receptor data (ER data)

The ER data were generated by Carroll [27], which mapped the association of the estrogen receptor on chromosomes 21 and 22. Here we just used a subset of the data to illustrate how the Bayesian latent model works. The subset we used is available from the BAC software [28]. It consists of intensity measurements for 30001 probes under the treatment and control conditions with three replicates each. The same subset has been used by BAC for a demonstration purpose.

**Figure 1:**
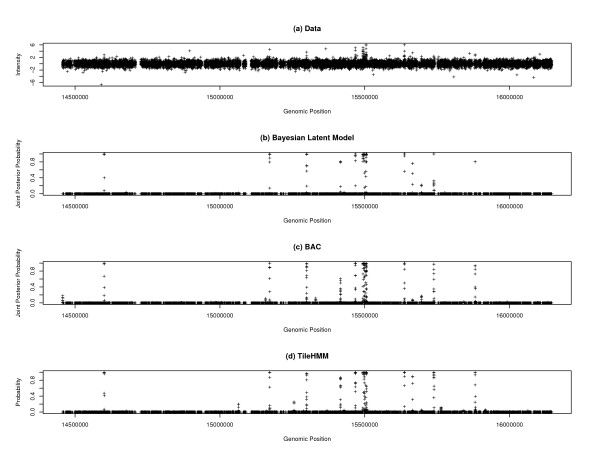
**Comparison results for the ER data**. (a) original data; (b) the joint posterior probability produced by the Bayesian latent model; (c) the joint posterior probability produced by BAC; and (d) the posterior probability produced by tileHMM.

The Bayesian latent model was first applied to the dataset. The algorithm was run 5 times. Each run consisted of 11000 iterations, and cost about 4.4 minutes CPU time on a personal computer (Intel Xeon 2.80 GHz, 1 G memory, Linux operating system). All computations of this paper were done on this computer. Based on the Gelman-Rubin diagnostic plot [29] [See Additional file 1 and 2], we discarded the first 1000 iterations for the burn-in process, and used the remaining 10,000 iterations for further inference. Figure 1(b) shows the estimates of the joint posterior probabilities resulted from one run.

For comparison, BAC and tileHMM (available at [30]) were also applied to this dataset. Both BAC and tileHMM produced a probability measure for each probe, similar to *ρ*_*i*_, on how likely it belongs to a bound region. The results were shown in Figures 1(c) and 1(d), respectively. The comparison shows that all the three methods produced very similar results for this dataset. However, the results produced by the Bayesian latent model are neater; the joint posterior probabilities produced by it tend to be dichotomized, either close to 1 or close to 0. This gives the user a clear classification for the bound and non-bound regions. To provide some numerical evidence for this statement, we calculated the ratio #{*i *: *P*_*i *_> 0.5}/#{*i *: *P*_*i *_> 0.05}, where #{*i *: *P*_*i *_> *a*} denotes the number of probes with *P*_*i *_greater than *a*. Here *P*_*i *_refers to the joint posterior probability for the Bayesian latent model and BAC, and the conditional probability for tileHMM. The ratios resultant from the Bayesian latent model, BAC and tileHMM are 0.816, 0.615 and 0.674, respectively.

Later, we assessed the sensitivity of the Bayesian latent method to the values of the hyperparameters *ν*_min _and *λ *with other parameters fixed, *α *= *β *= 0.05 and *ν*_max _= max_*i *_*y*_*i*_. The cross settings {0.5*s*_*y*_, 1.0*s*_*y*_, 1.5*s*_*y*_, 2*s*_*y*_, 2.5*s*_*y*_, 3*s*_*y*_} × [0.0001, 0.1] for (*ν*_min_, *λ*) were tried for this dataset. For each setting, the algorithm was run 5 times, and each run consisted of 11,000 iterations. To measure the similarity of the bound regions resultant from different settings of the hyperparameters, we propose to use the adjusted Rand index [31,32]. The adjusted Rand index is usually used in the literature of clustering, which measures the degree of agreement between two partitions of the same set of observations even when the comparing partitions having different numbers of clusters. It is obvious that the problem of bound region identification can also be viewed as a clustering problem; where the genome was partitioned into a series of segments, non-bound or bound regions, and each of the segments forms a cluster.

The adjusted Rand index is defined as follows. Let Ω denote a set of *n *observations, let *C *= {*c*_1_,...,*c*_*s*_} and  represent two partitions of Ω, let *n*_*ij *_be the number of observations that are in both cluster *c*_*i *_and cluster , let *n*_*i*_. be the number of observations in cluster *c*_*i*_, and let *n*._*j *_be the number of observations in cluster . The adjusted Rand index is(8)

A higher value of *r *means a higher correspondence between the two partitions. When the two partitions are identical, *r *is 1. When a partition is random, the expectation of *r *is 0. Under the generalized hyper-geometric model, it can be shown [32] that(9)

Refer to Hubert and Arabie [32] for the theoretical justification of *r*.

**Figure 2:**
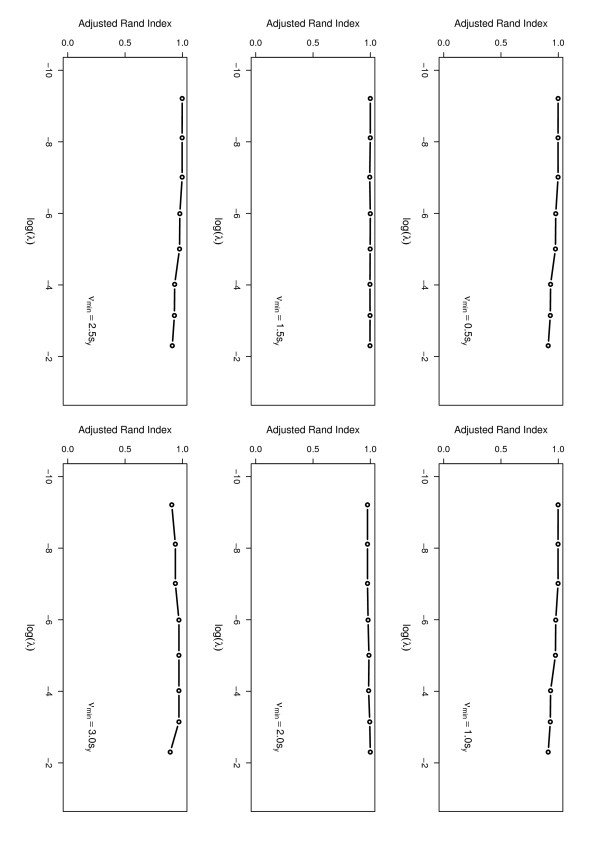
**Sensitivity analysis for the hyperparameters**.

In calculations of the adjusted Rand indices for the sensitivity experiments, we used the result shown in Figure 1(b) as the standard; that is, if a partition is identical to that partition, *r *will be 1. The results are summarized in Figure 2, where the adjusted *R*and index is plotted as a function of log(*λ*) for different setting of *ν*_min_. Figure 2 shows that, for each value of *ν*_min_, the adjusted *R*and index varies between 0.9 and 1.0 as *λ *runs from 0.0001 to 0.1. This indicates that the performance of the Bayesian latent model is rather robust to the choices of *ν*_min _and *λ*.

Finally, we examined the robustness of the Bayesian latent model to different choice of *w *and *m *with other parameters fixed at *α *= *β *= 0.05, *λ *= 0.01, and *ν*_*min *_= 2*s*_*y*_. The cross settings {2, 5, 7, 10} × {3, 5, 7} for (*w*, *m*) were tried for this dataset. Again, the adjusted *R*and index is used as the similarity criterion and the result shown in Figure 1(b) as the standard. The results were summarized in Table 1, which indicates, for this dataset, the Bayesian latent model is quite robust to the choices of *w *and *m*. In practice, to achieve robustness to outlying probes, we suggest to avoid choosing a small *m*. In all the following simulations, we set *m *= 5.

**Table 1:** Sensitivity analysis for the parameters *w *and *m*.

Adjusted Rand Index		*m*		
		
		3	5	7
*w*	2	0.987(0.006)	--	--
	5	0.987(0.006)	0.994(0.005)	0.839(0.020)
	7	0.991(0.005)	0.985(0.006)	0.834(0.010)
	10	0.994 (0.001)	0.987(0.007)	0.831(0.007)

The average of adjusted Rand indices (standard error in the parentheses) is calculated based on 5 independent runs. The entries for the cells with 2*w *<*m *are not available (--).

The robustness of the results with respect to changes of *α*, *β *and *ν*_*max *_are not studied in the paper. The reason is that *ν*_max _is completely determined by the data, and the values of *α *and *β *we used form a vague prior for the variance *σ*^2^.

### p53 data

In a ChIP-chip experiment, Cawley [1] mapped the binding sites of four human transcription factors Sp1, cMyc, p53-FL, and p53-DO1 on chromosomes 21 and 22. The experiment consisted of 6 treatment and 6 input control arrays, and the chromosomes spanned over three chips *A*, *B *and *C*. Refer to Cawley [1] for the details of the experiment. For the testing purpose, p53-FL data on chips A, B and C were used in this paper, which contains 14 quantitative PCR verified regions. As in Cawley [1], the data were pre-processed by filtering out the local repeats, quantile-normalized [33], rescaled to have a median feature intensity of 1000 for the purpose of adjusting batch effect, and then log-transformed. Since the normalization is not the focus of this paper, we skipped the details.

The Bayesian latent method was first applied to the p53 data. The data on chip A, chip B, and chip C were analyzed separately. Each run consisted of 11,000 iterations. Diagnostic plot for the convergence of these runs indicates that they can converge within several hundreds of iterations, even the data on each chip consists of more than 300,000 probes. Accordingly, the first 1000 iterations were discarded for the burn-in process, and the samples from other iterations are used for further analysis.

For comparison, BAC and tileHMM were also applied to this example. Given the posterior probabilities, a cutoff of 0.5 was used for all methods to detect bound regions. All resultant bound regions having less than 3 probes or 100 bps were considered to be spurious and removed, and those regions separated by 500 bps or less were merged together to form a predicted bound regions following the approach taken by Cawley [1]. The results were summarized in Table 2. Although tileHMM detected all the 14 validated regions, it essentially fails for this example. It identified a total of 33796 bound regions, which should contain too many false bound regions. We suspect that the failure of tileHMM for this example is due to its training algorithm; it is very likely that tileHMM converged to a local maximum of the likelihood function. This have been noted by Humburg et al. [10], tileHMM may converge to a local maximum of the likelihood function with either the Baum-Welch algorithm or the Viterbi training algorithm, rendering an ineffective inference for the model.

**Table 2:** Computational results for the p53-FL data with a cutoff of 0.5.

	Chip A		Chip B		Chip C		p53	
				
Method	V(2)	Total	V(3)	Total	V(9)	Total	V(14)	Total
Bayesian latent	2	15	2	28	8	27	12	70 (127)
BAC	2	38	1	29	9	33	12	100 (1864)
tileHMM	2	29708	3	1944	9	2144	14	33796

Both the total numbers of regions and quantitative PCR verified(V) ones detected by each method on each chip are reported. The columns under "p53" summarize the results on chips A, B and C. The number in the parentheses is the number of clusters needed to cover all 14 experimentally validated bound regions.

Both the Bayesian latent method and BAC work well for this example. At a cutoff of 0.5, BAC identified 100 bound regions, which cover 12 out of 14 experimentally validated bound regions. The Bayesian latent method works even better. At the same cutoff, it only identified 70 bound regions, but which also cover 12 out of 14 experimentally validated bound regions. For further comparison of the Bayesian latent method and BAC, we relaxed the cutoff value and counted the total number of regions needed to cover all experimentally validated regions. We found that the Bayesian latent method only needs to increase the total number of regions to 127, while BAC needs to increase to 1864 regions. Note that the BAC and tileHMM's results reported here may be a little different from those reported by other authors, due to the difference of the normalization methods.

### Simulated data

To have a careful assessment of the performance of the Bayesian latent model, we simulated 10 datasets based on the Sp1 data of Cawley's [1] experiment. Each dataset consists of 200,000 probes, two conditions (control and IP-enriched), and six replicates under each condition. The probe genomic coordinates we used in simulations were the first 200,000 genomic positions used in the Sp1 data. Each dataset consisted of 996 bound probes, forming 50 bound regions. As in Gottardo [13], the bound regions were assumed to describe a peak with the intensity function given by *A *exp{-4(*g*_*i *_- *C*)^2^/*B*^2^}, where *A *is the amplitude of the peak, *B *controls the width of the peak, *C *represents the center of the peak, and *g*_*i *_is the genomic position of probe *i*. We also followed Gottard [13] to generate the centers of the bound regions randomly across the set of possible coordinates while imposing a separation of at least 3000 bps between peaks; and to generate the values of parameter *B *uniformly between 600 and 1000 bps. The values of parameter *A *were generated uniformly between 3 and 5. The variance of the probe intensity was estimated from the Sp1 data.

**Figure 3:**
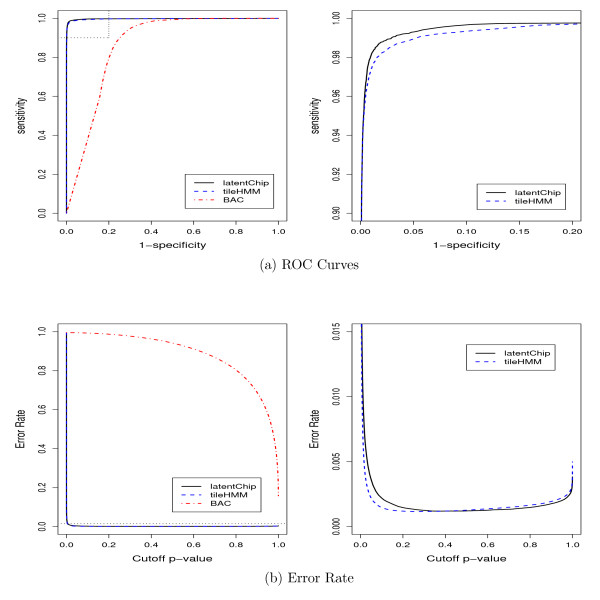
**Averaged ROC curves and error rates for different models on simulated datasets**. (a) ROC curve; (b) error rate. All the plots were obtained by averaging over the results for the 10 datasets. The plots on the right provide a closer view for the area enclosed by the dotted line and axis on the left.

Firstly, the performance of different models is assessed using the area under the receiving operating characteristic (ROC) curve [34] and the error rate. The former is a standard measure for the performance of a multiple hypothesis testing method, which shows the true positive discovery rate (*sensitivity*) against the false positive discovery rate (1 - *specificity*) at probe level. The later is a standard measure for the performance of a classification method, which shows the proportion of totally incorrect probe calls, including both false positives and false negatives, against different cutoff values. All the three methods, Bayesian latent method, BAC and tileHMM, were applied to the 10 full datasets. The averaged ROC curve and error rate across a range of cutoffs are obtained and plotted in Figure 3. As indicated by Figure 3(a), the Bayesian latent method and tileHMM have very similar performances on these datasets, and both are much better than BAC. By further examining the plot on the right, which provides a closer view of the area enclosed by the dotted line and axis on the left, it is easy to see that the Bayesian latent method is better than tileHMM for this example. Next, we checked the error rate for each model. The results were shown in Figure 3(b). Again, the Bayesian latent method and tileHMM perform very well and both are much better than BAC. From the right plot of Figure 3(b), we can see that the optimal cutoff for tileHMM is close to 0.3, while it is close to 0.5 for the Bayesian latent method. Figure 3(b) also suggest that both the Bayesian latent method and tileHMM are robust to the choice of cutoff values, ranging from 0.2 to 0.8, while BAC is not.

Later, based on the true bound regions which are known for these 10 simulate datasets, we use the adjusted Rand index *r *to assess the quality of the results produced by the above three algorithms. In addition, we calculated *p*-values of the two-sample *t*-tests, *H*_0_: *r*_*BL *_= *r*_*O *_*vs H*_1_: *r*_*BL *_> *r*_*O*_, where *r*_*BL *_denotes the *r*-value produced by the Bayesian latent model, and *r*_*O *_denotes the *r*-value produced by the other method. The results were summarized in Table 3. The tests indicate that the Bayesian latent model can lead to more accurate identifications of true bound regions than BAC and tileHMM.

**Table 3:** Computational results for the simulated datasets.

Method	Total	ND	FD	*r*	***p*-value**
Bayesian Latent	50.5 (0.58)	2.3 (0.33)	2.8 (0.57)	0.9545 (0.0080)	--
tileHMM	48 (0.77)	4.2 (0.57)	2.2 (0.55)	0.9250 (0.0107)	0.02
BAC	2934.7 (6.60)	0 (0)	2884.7 (6.6)	0.0609 (0.0003)	0.00

Wilcox	56.1 (0.95)	3.9 (0.48)	6.4 (0.62)	0.9221 (0.0088)	0.007
*t*-scan	78.9 (2.11)	3.1 (0.31)	27.6 (1.71)	0.9047 (0.0089)	0.0003
EB *t*-Scan	71.5 (1.52)	3.0 (0.39)	20.9 (1.38)	0.9176 (0.0068)	0.001

"Total" denotes the average number of bound regions identified for each of the 10 datasets, *ND *denotes the number of true bound regions that are not discovered by the algorithm, *FD *denotes the number of false bound regions discovered by the algorithm, *r *is the adjusted Rand index, the number in the parentheses is the standard error, and "EB *t*-scan" refers to the empirical Bayesian *t*-scan method proposed by Ji and Wong [6].

For a thorough comparison, we also applied the sliding window methods, including the Wilcoxon rank sum test method [1], *t*-scan statistic [7] and empirical Bayesian *t*-scan statistic [6], to the 10 datasets. For the testing purpose, we identified the most significant 996 probes, which is the same as the true number of bound probes, as the bound probes for each of the datasets and each of the sliding window methods. We note that this cutoff number should be determined by a multiple hypothesis test in practice, and this choice makes the comparison a little favorly biased toward the sliding window methods. The results were summarized in the lower panel of Table 3, which indicate that the Bayesian latent model also outperforms the sliding window methods.

## Discussion

The Bayesian latent model can be generalized in a few ways. Firstly, it can be generalized to allow different bound regions to have different variances. This generalization has been implemented by us. The numerical results are very similar to those reported in the paper.

Secondly, it can be generalized to work on the multiple replicates directly. This can be simply done by modifying (2) to multivariate normals. This generalization will certainly slow down the simulations, but the results may not be improved significantly. The reason is that under the assumption of constant variances for probe intensities, the statistic (1) is sufficient for the mean intensity of probes, while the latter has been designed in the experiment as the main measure for differentiating bound and non-bound regions.

The reason why the Bayesian latent method outperforms tileHMM and BAC can be explained as follows, through a detailed comparison of the models used by them. TileHMM implemented a standard two-state hidden Markov model, with the emission distribution of state *S*_*i*_, *i *= 1, 2, being modeled as a *t*-distribution. TileHMM and the Bayesian latent model are mainly different in two respects.

• TileHMM is a non-Bayesian method, where maximum likelihood estimates are used for all model parameters and inference for the bound regions are based on the conditional probability of the hidden states. TileHMM is trained using the Baum-Welch algorithm and the Viterbi algorithm. It is known that the Baum-Welch algorithm is an EM algorithm implemented in the context of HMM, and that it tends to converge to a local maximum of the likelihood function. The Viterbi algorithm provides a fast alternative to the Baum-Welch algorithm, but may not converge to a local maximum. The Bayesian latent method is a Bayesian method, where inference for bound regions is based on the posterior distribution of the latent variable. The posterior distribution is simulated using the Metropolis-within-Gibbs sampler, which is known to converge to its target distribution when the number of iterations becomes large.

• TileHMM models all bound regions to have the same mean value, while the Bayesian latent model allows different bound regions to have different mean values. Our model fits the real data better.

The mixed performance of tileHMM on the simulated and real datasets indicates that the inferiority of tileHMM is mainly due to its training algorithm. In addition, as indicated by our simulated examples, tileHMM tends to misidentify the bound regions with relatively low probe intensities, because it models all bound regions to have the same mean value.

BAC models the probe intensity using a mixed-effect model:

where *c *= 1 denotes the control sample, *c *= 2 denotes the treatment sample, *r *is the index of replicates; *μ*_*p *_is a random probe effect distributed as ; *γ*_*cp *_is the probe enrichment effect with *γ*_1*p *_= 0; and ϵ_*cpr *_is the random error distributed as . The authors further modeled the probe enrichment effect by a mixture of a point mass at zero and a truncated Gaussian distribution, i.e.,

where *TN*_+_() denotes a truncated Gaussian distribution truncated at zero, and *w*_*p *_is the *a priori *proportion of probes belonging to nonbound regions. The *a priori *proportion depends on a latent Markov random field prior ***θ ***= {*θ*_*p*_, 1 ≤ *p *≤ *P *}, through a logistic transformation

and a Gaussian intrinsic autoregressive model [18] for ***θ***,

where ∂_*p *_corresponds to the probes *p' *immediately adjacent to *p*, *n*_*p *_is the cardinality of ∂_*p*_, *κ *is a smoothing parameter, and *n *is the number of neighboring probes used. The model is trained using a MCMC algorithm.

The main difference between the BAC and the Bayesian latent methods is that BAC models the control and treatment samples jointly, while the Bayesian latent method models the difference between the averaged treatment and control samples. Since BAC models the treatment and control samples jointly, it has to include the probe-specific effect in the model and assume a complicated structure for the random error, assuming the variance depends on both the probe and the type of samples (control or treatment). By working on the difference between the averaged treatment and control samples, the Bayesian latent method eliminates the probe effect in the model and the dependence of the random error on the probe and the type of samples. This simplifies the model greatly and enables an efficient MCMC simulation from the the posterior distribution. In addition, due to the complicated structure of the model, BAC includes too many parameters, and this makes the model potentially overfitted, especially when the number of replicates is small. This explains why BAC always tends to identify too more bound regions than does the Bayesian latent model. On the other hand, the simplicity of the Bayesian latent model makes it rather robust to outlying probes. As indicated by our examples, it work well for all examples studied in this paper.

## Conclusion

We have proposed a Bayesian latent model for the ChIP-chip experiments. The new model mainly differs from the existing Bayesian models, such as the joint deconvolution model, the hierarchical gamma mixture model, and the Bayesian hierarchical model, in two respects. Firstly, it works on the difference between the averaged treatment and control samples. This enables the use of a simple model for the data, which avoids the probe-specific effect and the sample (control/treatment) effect. As a consequence, this enables an efficient MCMC simulation of the posterior distribution, and also makes the model fairly robust to the outliers. Secondly, it models the neighboring dependence of probes by introducing a latent indicator vector. A truncated Poisson prior distribution is assumed for the latent indicator variable, with the rationale being justified at length.

The Bayesian latent model has been successfully applied to the ER, p53, and some simulated datasets, with comparisons with BAC, tileHMM, and some sliding window methods. The numerical results indicate that the Bayesian latent model can outperform others, especially when the dataset contains outlying probes.

## Availability and requirements

An **R **software package called **LatentChIP**, which implements the Bayesian latent model under linux operating system, is available at the author's web-page [35].

## Authors' contributions

MW developed the **R **software package, analyzed the data, and drafted the paper. FL conceived this work, developed the model, and drafted the paper. YT participated in the discussion of ChIP-chip technology. All authors read and approved the final manuscript.

## Appendix

The scheme for simulating samples from the posterior distribution:

(a) Conditioned on *z*^(*t*)^, updating  using the Metropolis-Hastings (MH) algorithm, where *t *indexes the number of iteration cycles.

(b) Conditioned on , updating each component of z^(*t*) ^according to the following rule: Given : change  to  using the MH algorithm.

When a component of *z *is updated in step (b), the sum of square terms in the posterior density function can be calculated in a recursive manner, i.e., only the terms related to *z*_*i *_need to be re-calculated.

## Supplementary Material

Additional file 1 **Convergence study of Bayesian latent model**. This file provides a convergence study of Bayesian latent model for the ER dataset. Click here for file

Additional file 2 **Comparison results for simulated data**. This file provides a comparison study for BAC, tileHMM and the latent model on simulated data. Click here for file
