# Supplementary material for: Bayesian modeling of ChIP-chip data using latent variables
Source: BMC Bioinformatics. 2009 Oct 26;10:352. doi: 10.1186/1471-2105-10-352 (PMC2779819; doi:10.1186/1471-2105-10-352)
Supplement: Additional file 1 — Convergence study of Bayesian latent model. This file provides a convergence study of Bayesian latent model for the ER dataset. [file 1471-2105-10-352-S1.PDF]

Bayesian modeling of ChIP-chip data using latent variables
Additional file 1

Mingqi Wu, Faming Liang and Yanan Tian

Convergence study of Bayesian latent model

The Bayesian latent model was applied to the ER dataset. The algorithm was run 5 times. Each run consisted of 11000 iterations. Figure 1 provides a diagnostic plot for the convergence of the runs, where the statistic Gelman-Rubin $\hat{R}$ (Gelman and Rubin 1992) was plotted versus iterations. The simulations are usually considered to be converged when the statistic Gelman-Rubin $\hat{R}$ falls below the horizontal line 1.1. Figure 1 indicates that for this example, the simulations converged very fast, usually within two hundreds of iterations.

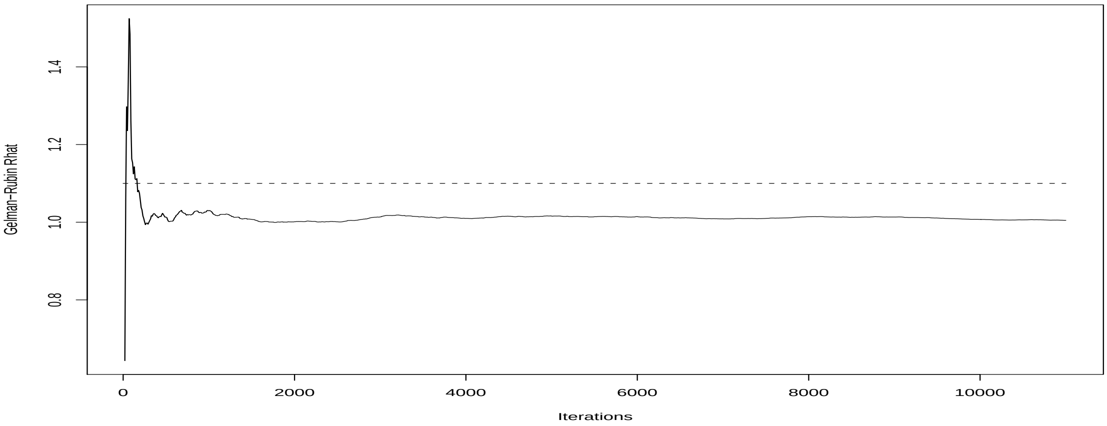

Figure 1: Convergence diagnostic of the Bayesian latent method for the ER example

.
