# Supplementary material for: Bayesian modeling of ChIP-chip data using latent variables
Source: BMC Bioinformatics. 2009 Oct 26;10:352. doi: 10.1186/1471-2105-10-352 (PMC2779819; doi:10.1186/1471-2105-10-352)
Supplement: Additional file 2 — Comparison results for simulated data. This file provides a comparison study for BAC, tileHMM and the latent model on simulated data. [file 1471-2105-10-352-S2.PDF]

Bayesian modeling of ChIP-chip data using latent variables
Additional file 2

Mingqi Wu, Faming Liang and Yanan Tian

Comparison results for simulated data

For the simulated data, Figure 1 (a) shows the intensity values of the first 20000 probes, as processed in (1).

$$Y_i = \bar{X}_{1i} - \bar{X}_{2i}, \tag{1}$$

where $\bar{X}_{li}$ is the intensity measurement of probe i averaged over m_l replicates. The data seems a little noisy, but still contains enough information for identification of the true bound regions. To see this, we first smoothed each replicate of the data using a moving window approach with a window size of 1000bp, and then calculated the smoothed intensity values as in (2).

$$\widetilde{Y}_i = \widetilde{X}_{1i} - \widetilde{X}_{2i}, \tag{2}$$

where $\widetilde{X}_{li}$, $l = 1, 2$, denotes the average of the smoothed data under condition l. Figure 1 (b) plots the smoothed data, where the five bars, centered at 681, 5188, 8293, 13122 and 16145, correspond to the five true bound regions, respectively. To make the problem more challenging, we tested our algorithm on the non-smoothed data. Our algorithm was run for 11000 iterations, for which the first 1000 iterations were discarded for the burn-in process, and the remaining 10000 iterations were used for inference. Figure1 (c) showed the estimates of the joint posterior probabilities. It indicate that the five bound regions have been identified by our algorithm accurately. For comparison, BAC and tileHMM were also applied to the same dataset, with the results being shown in Figures 1(d)&(e), respectively. The comparison shows that BAC totally fails for this datasets; and tileHMM works for the dataset, but the bound regions identified by it tend to be falsely prolonged. This example suggests that the Bayesian latent model is more robust to outlier probes than BAC and tileHMM.

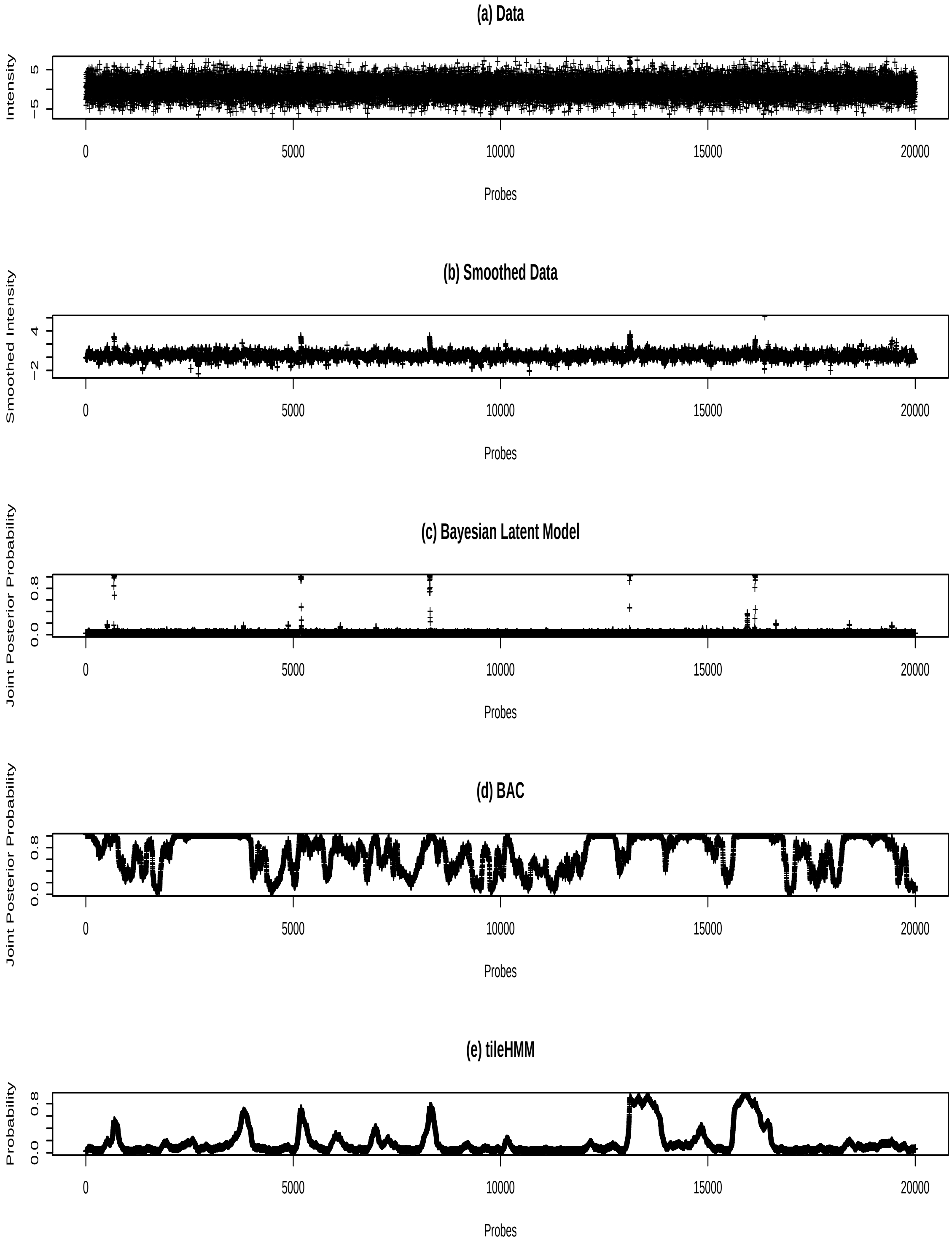

Figure 1: Comparison results for the simulated data: (a) non-smoothed data; (b) smoothed data; (c) output of the Bayesian latent model; (d) output of BAC; and (e) output of tileHMM;
